# The machine learning assisted optimization of ultrasound-synergistic enzymatic extraction and comprehensive evaluation of polysaccharides from *Saposhnikovia divaricata*

**DOI:** 10.1016/j.ultsonch.2025.107706

**Published:** 2025-12-06

**Authors:** Zeyu Li, Qian Li, Chaogui Hu, Feng Yang, Fengxia Guo

**Affiliations:** a College of Agronomy, State Key Laboratory of Aridland Crop Science, Gansu Agricultural University, Lanzhou 730070, China; b College of Life Science and Technology, Gansu Agricultural University, Lanzhou 730070, China

**Keywords:** *Saposhnikovia divaricata*, Polysaccharide, Ultrasound-synergistic enzymatic, Machine learning, Antioxidant

## Abstract

*Saposhnikovia divaricata* (*S. divaricata*) is a common medicinal material with remarkable medicinal value, but there is limited research on its polysaccharides. This research employed ultrasound-synergistic enzymatic extraction to obtain polysaccharides, while leveraging response surface methodology (RSM) and support vector regression (SVR) models to optimize the extraction procedure. According to the SVR model, the optimal extraction conditions that led to a superior polysaccharide yield of 11.82 % included maintaining a solid-solvent ratio of 1:13 g/mL, conducting the process at an extraction temperature of 45 °C with 420 W of power, adjusting the pH to 4.5, extending the ultrasonic treatment to 35 min, and incorporating a 3 % enzyme dosage. Additionally, a comparative analysis of polysaccharides extracted through different methods assessed their physicochemical characteristics, structure, and antioxidant capacity. The entropy weight method was employed for a comprehensive evaluation of the different extraction techniques. In the final assessment, the ultrasound- synergistic enzymatic extraction emerged as the top-performing technique, achieving the highest composite score. Notably, the antioxidant efficacy was driven primarily by the levels of uronic acid, molecular weight and mannose. This research establishes the theoretical foundation and scientific rationale for the efficient extraction and bioactive application of *S. divaricata* polysaccharides (SDP).

## Graphical abstract

**Figure:**
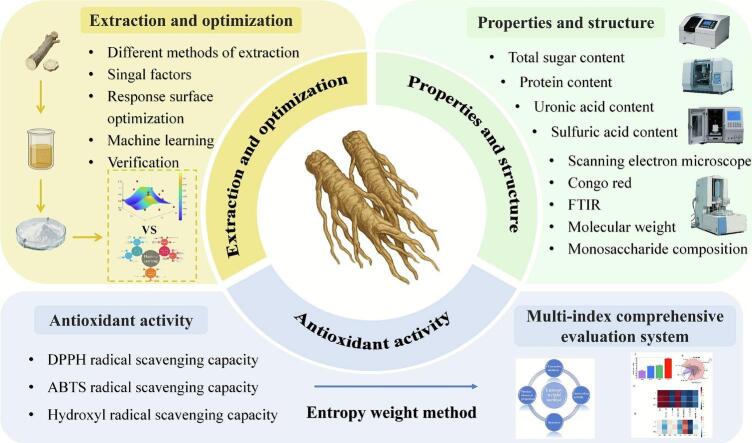

## 1 Introduction

*Saposhnikovia divaricata (S. divaricata)* is the dried root of the plant, *Saposhnikovia divaricata* (Turcz.) Schischk, belonging to the Apiaceae family [1]. Thriving in arid grasslands of Northeast Asia, this highly adaptable plant exhibits notable tolerance to cold, drought, and alkaline conditions [2,3]. With a long history of medicinal use, *S. divaricata* is valued for its efficacy in dispelling wind and alleviating exterior syndromes, eliminating dampness and alleviating pain, and relieving spasm. It is mainly indicated for exogenous wind-cold, headache, blurred vision, stiff nape, wind-cold-dampness arthralgia, and other symptoms [4,5]. Volatile oils, chromones, coumarins, polysaccharides, and organic acids represent the principal chemical constituents of *S. divaricata* [6]. Pharmacological studies have shown that *S. divaricata* exerts a broad range of bioactivities, including antipyretic, analgesic, anti-allergic, anticonvulsant, antitumor, immunomodulatory, and antioxidant effects [[7], [8], [9], [10], [11], [12]]. For a long time, scholars have conducted extensive analytical studies on the structures and activities of small-molecule compounds in *S. divaricata*, such as coumarins and chromones. However, a detailed exploration of the structure and bioactivity of *S. divaricata*'s natural macromolecular polysaccharides is relatively limited. Natural polymeric macromolecules, classified as polysaccharides, are recognized for their extensive biological functions. Studies have demonstrated that polysaccharides possess multiple effects, including anticancer, antitumor, immune enhancement, blood glucose control, antioxidant, and anti-aging effects [[13], [14], [15]]. Therefore, achieving efficient utilization of polysaccharides has become an important issue that urgently requires attention in the relevant research fields.

The extraction methods applied to polysaccharides are pivotal in shaping their molecular architecture and influencing their biological activities. At present, polysaccharides are primarily obtained through several extraction approaches, including hot-water extraction, dilute acid or alkali extraction, enzyme-mediated processes, as well as techniques based on microwave irradiation and ultrasonic assistance [16,17]. Owing to its simplicity, hot-water extraction is still the standard approach, yet it is constrained by long processing times and inadequate cost efficiency [18]. In practical polysaccharides extraction processes, two or more extraction methods may be used synergistically to obtain polysaccharides components with higher extraction yields and activity [19]. Among these techniques, ultrasonic extraction exploits the mechanical, cavitation and thermal effects of ultrasound to disrupt plant cell-wall structures, thereby facilitating the dissolution of intracellular active components and ultimately achieving efficient polysaccharide extraction [20]. In addition, enzymatic extraction employs cellulase, pectinase and papain to hydrolyze plant cells as well as pectin and glycoprotein complexes, resulting in the release of greater amounts of polysaccharides [21,22]. Ultrasound-synergistic enzymatic extraction represents an integrated approach that synergizes the benefits of both ultrasonic and enzymatic methods. It provides ease of operation, high productivity, minimal energy usage, gentle processing, and eco-friendliness [23,24].

Optimizing extraction processes for active components in natural products remains a crucial research direction for maximizing resource utilization. Response Surface Methodology (RSM) is widely used in process optimization research. Through experimental design, model construction, and quadratic polynomial equation fitting, it assesses how independent variables and their interactions influence the responses [25]. In recent years, significant achievements have been obtained in optimizing extraction process variables through the application of RSM [23,26,27]. However, RSM exhibits limitations when handling highly nonlinear, high-dimensional coupled systems. Its reliance on predefined model structures restricts its ability to characterize complex response relationships, making it difficult to fully capture the deep characteristics between variables. Consequently, incorporating machine learning (ML) methods has emerged as an effective strategy to enhance model performance and prediction accuracy. As a core component of artificial intelligence, ML autonomously learns intrinsic patterns in data through data-driven approaches and is well-suited for modeling and optimizing multidimensional, nonlinear, complex tasks. Support Vector Regression (SVR) demonstrates particular advantages when handling small sample sizes and nonlinear regression problems [[28], [29], [30]]. Kernel functions in SVR effect a high-dimensional embedding of the data, facilitating precise nonlinear fitting while ensuring stable generalization and resistance to perturbations. Consequently, ML techniques (particularly SVR) have been progressively applied to modeling and optimizing bioactive compound extraction processes [31,32]. They demonstrate superior predictive performance and optimization efficiency compared to traditional RSM methods, providing powerful tools for in-depth research in this field. In their investigation into extracting compounds from mulberry leaves, scholars discovered that the SVR model outperformed RSM by delivering more precise predictions, tighter alignment between theoretical and actual results, and a clearer representation of extraction efficiency. The data clearly favored SVR approach, showcasing its superior reliability in modeling the process [33].

The components of traditional Chinese medicine (TCM) are complex, and their efficacy often results from the synergistic action of multiple components. A single indicator is difficult to fully reflect its quality or process applicability. Therefore, it is necessary to integrate multiple indicators such as physicochemical properties, active component content, and biological activity for comprehensive evaluation. The entropy weight method can transform complex multi-indicator data into a single comprehensive evaluation value through the standardized processing and weighted summation of multi-dimensional indicators. It is able to intuitively rank the quality of medicinal materials from different origins and products from different extraction processes. This technology has seen widespread use across multiple domains, including the optimization of extraction processes for TCM and its compound preparations, the assessment of quality grades for medicinal materials, and the screening of bioactive components [34,35], providing a reliable quantitative tool for the “multi-indicator-multi-level” comprehensive decision-making in TCM research.

In this study, polysaccharides from *S. divaricata* were extracted by ultrasound-synergistic enzymatic treatment. Cellulase, pectinase, and papain were used to target and hydrolyze different components of plant cell structure (cellulose, pectin, and protein). The extraction process optimization was achieved through the application of RSM and ML, achieving efficient and gentle extraction of polysaccharides from *S. divaricate*. Additionally, the study compared polysaccharides from *S. divaricata* extracted via various methods, analyzing their physicochemical characteristics, structural features, and antioxidant capabilities. On this basis, a multi-index comprehensive evaluation system was further established using entropy weight method to screen the optimal extraction method of SDP, and specific indicators affecting the antioxidant activity of SDP were determined, establishing conceptual foundations for applying SDP.

## 2 Materials and methods

### 2.1 Materials and chemicals

*S. divaricata* pieces (production batch No. 240501) were purchased from Beijing Tongrentang (Beijing, China). Glucose and glucuronic acid standards were obtained from Sigma-Aldrich (St. Louis, MO, USA). Trichloroacetic acid, anhydrous ethanol, phenol, sulfuric acid, Coomassie brilliant blue reagent, bovine serum albumin, potassium sulfate standard, hydrochloric acid, barium chloride, gelatin, carbazole, iodine, potassium iodide, Congo red, ferrous sulfate, salicylic acid, hydrogen peroxide and sodium hydroxide were all of analytical grade and purchased from Sinopharm Chemical Reagent Co., Ltd. (Shanghai, China). DPPH (1,1-diphenyl-2-pic­ rylhydrazyl), ABTS^+^ (2,2′-azino-bis(3-ethylbenzothiazoline-6-sulfonic acid)), cellulase, pectinase, and papain were purchased from Macklin Biochemical Tech­ nology Co., Ltd (Shanghai, China). Deionized water was used throughout all experiments.

### 2.2 Material pre-treatment

The processed slices of *S. divaricata* were oven-dried at 40℃ until reaching constant weight crushed and sieved, subjected to reflux degreasing to remove impurities and small molecular substances, dried, and then set aside for use.

### 2.3 Extraction of SDP

1.0 g of *S. divaricata* powder was weighed and treated with hot-water reflux, enzyme treatment, ultrasonic treatment, and ultrasound-synergistic enzyme treatment to obtain SDP.

Hot water extraction of polysaccharides (HWP): *S. divaricata* powder was combined with deionized water at a ratio of solid-solvent 1:10 g/mL, and the mixture was subsequently refluxed for 1.5 h. After retaining the filtrate, the remaining residue was combined with a suitable amount of deionized water and refluxed again for 1.5 h. The combined filtrates were centrifuged to remove residual impurities and subsequently concentrated under reduced pressure to one-third of the initial volume, after which four volumes of absolute ethanol were added for precipitation. After allowing the mixture to stand overnight, the precipitate was harvested via centrifugation. It was then re-dissolved in distilled water and combined with an equal volume of 10 % trichloroacetic acid. The resulting solution was shaken for 10 min. The mixture was stored at 4 °C for 30 min, and the protein precipitate was subsequently isolated through centrifugation. The dialysis tubing was cut into 15–30 cm segments and activated in a boiling water bath for 5 min, followed by thorough rinsing with distilled water before use. The deproteinized polysaccharides solution was dialyzed against running water using a 3500 Da dialysis membrane for 12 h. After dialysis, the solution was concentrated to obtain the polysaccharides solution with small molecules removed. Absolute ethanol was introduced into the supernatant, and the mixture was allowed to precipitate at 4 °C for 12 h. The resulting precipitate was collected by centrifugation and subsequently freeze-dried to obtain SDP.

Ultrasound-synergistic water extraction of polysaccharides (UWP): Ultrasonic parameters were adjusted to 30 min for duration, 50 ℃ for extraction temperature, and 240 W for power. The centrifugation and alcohol precipitation procedures were performed as described previously, resulting in the collection of the SDP.

Enzymatic-water extraction of polysaccharides (EWP): After adding 2 % enzyme, the solution was adjusted to pH 5.0 and heated in a water bath at 50 °C for 30 min, followed by deactivation at 100 ℃. Centrifugation and alcohol precipitation were performed as described above to obtain EWP.

Ultrasound-synergistic enzyme water extraction of polysaccharides (UEWP): The ultrasonic process was performed for 30 min at 50 ℃ with a power of 240 W. Afterward, 2 % enzyme was added, the pH was adjusted to 5.0, inactivate, centrifuge, and centrifugation followed by alcohol precipitation was carried out as previously described to obtain UEWP.

The dried polysaccharide calculated according to the Eq. (1) [36].

(1) $$\begin{array}{c} Yield\left(\%\right)=\frac{m_{1}}{m_{2}}\times 100\% \end{array}$$

where *m_1_* is the mass of polysaccharides, and *m_2_* is the mass of *S. divaricata* powder.

### 2.4 Single-factor experiments

Based on the best extraction method, the extraction was optimized with solid-solvent ratio (1:5, 1:10, 1:15, 1:20 and 1:25 g/mL), ultrasonic time (20, 25, 30, 35, 40 min), extraction temperature (35, 40, 45, 50, 55 °C), ultrasonic power (240, 300, 360, 420, 480 W), pH (4, 4.5, 5, 5.5, 6) and enzyme dosage (based on medicinal material powder) (1 %, 2 %, 3 %, 4 %, 5 %) as single factors.

### 2.5 Plackett-Burman (PB) design

To further identify the significant influencing factors, Design-Expert 13.0 was used to conduct a PB experimental design. Two levels (−1, 1) were selected for each influencing factor in the single-factor experiment. The levels corresponding to each factor are summarized in Table 1.

**Table 1:** Factors and levels of PB design.

**Levels**	**Solid-solvent ratio** **(g/mL)**	**Ultrasonic power** **(W)**	**Extraction temperature** **(℃)**	**Ultrasonic time** **(min)**	**pH**	**Enzyme dosage** **(%)**
−1	1:5	240	35	20	4	1
1	1:25	480	55	40	6	5

### 2.6 Optimizing extraction with RSM

The solid–solvent ratio (A), extraction temperature (B), and pH (C) were chosen as RSM variables, as determined from single-factor and PB experimental results. Ultrasonic power was maintained at 420 W, ultrasonic time at 35 min, and enzyme dosage at 3 %. The extraction process was optimized using a Box-Behnken design (BBD), taking the comprehensive polysaccharide yield (Y) as the response variable. Each factor was evaluated at three levels: high (1), medium (0), and low (−1). The corresponding variable levels are shown in Table 2.

**Table 2:** Factors and levels of BBD.

**Factors**	**Level**		
	**−1**	**0**	**1**
Solid-solvent ratio (g/mL)	1:10	1:15	1:20
Extraction temperature (℃)	40	45	50
pH	4.5	5	5.5

### 2.7 Machine learning (ML) modeling

**Fig. 1:**
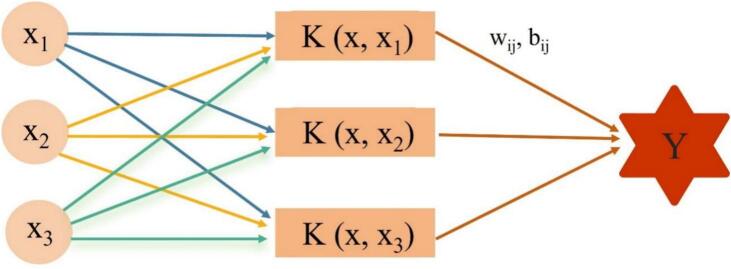
Schematic diagram of SVR model structure. (Note: w_ij_ represents the weight coefficients of the decision function, b_ij_ represents the bias term of the decision function).

To establish a nonlinear mapping relationship between ultrasound-synergistic extraction process parameters (solid-solvent ratio, extraction temperature, and pH) and the target value (yield) and to identify the optimal parameter combination, a SVR integrated modeling and optimization strategy using Bayesian optimization combined with a genetic algorithm was employed. Deep modeling of data based on RSM design points was performed. In the model, X represents the input variable (1, 2, and 3 represent solid-solvent ratio, extraction temperature, and pH, respectively), and Y (yield) represents the output variable (Fig. 1). To eliminate dimensional differences among parameters and improve model convergence speed and performance, data preprocessing was performed using standardization. Input features were scaled to the [0, 1] range using min–max scaling, and the output variable (yield) was normalized using the Z-score method. To automatically identify the globally optimal hyperparameter combination, a Bayesian optimization combined with a genetic algorithm framework was employed. Model training and evaluation were performed 200 times using a minimization 5-fold cross-validation approach. Model performance was assessed comprehensively using the coefficient of determination (R^2^), root mean square error (RMSE), mean square error (MSE), and mean absolute error (MAE) [37]. All computations were carried out in the MATLAB R2021a environment.

### 2.8 Determination of physicochemical properties of SDP

Following the methods of previous researchers [[38], [39], [40], [41]], the total sugar, protein, uronic acid, and sulfate content of SDP were determined. The experimental methods are detailed in Supplementary information S1.1–1.4.

### 2.9 Structural characterization of SDP

#### 2.9.1 Scanning electron microscope (SEM)

The dried polysaccharide was sprayed with gold and then placed under a scanning electron microscope (S3400N, Hitachi, Tokyo, Japan) at 5.00 kV and a magnification of 1500 × to observe its morphological characteristics, and the image was photographed.

#### 2.9.2 Fourier transform infrared spectroscopy (FTIR) scanning

A 1.0 mg sample of polysaccharide was ground with KBr, which had been dried to constant weight, in a mass ratio of 1:100. After grinding and tableting, the sample was scanned in the range of 4000–400 cm^−1^ using an infrared spectrometer (FTIR-650, Gangdong, China).

#### 2.9.3 Congo red analysis

To characterize triple-helix structures in the polysaccharides, a Congo red test was conducted [42]. According to Zhang et al. [43], polysaccharide solutions were mixed 1:1 (*v/v*) with Congo red, followed by supplementation with NaOH at graded concentrations. Spectra (400–600 nm) were acquired and analyzed using a UV–Vis instrument (UV-2450, Shimadzu, Japan). Full experimental details are available in Supplementary information S1.5.

#### 2.9.4 Molecular weight determination

The analysis of SDP was performed using a Gel Permeation Chromatography system coupled with Refractive Index and Multi-Angle Light Scattering detectors. This system consisted of a Thermo (USA) U3000 liquid chromatography unit, a Wyatt Technology (CA, USA) Optilab T-rEX differential refractometer, and a DAWN HELEOS II multi-angle laser light scattering detector. For detailed experimental parameters, please refer to Supplementary information S1.6.

#### 2.9.5 Monosaccharide composition determination

The chromatographic analysis was performed on a Thermo ICS 5000 + ion chromatography system (Thermo Fisher Scientific, USA) equipped with an electrochemical detector for the determination of monosaccharide composition. For detailed experimental parameters, please refer to Supplementary information S1.7.

### 2.10 Determination of antioxidant activity of SDP in vitro

The antioxidant activity of SDP was evaluated using DPPH, ABTS^+^, and hydroxyl radical assays following the procedure reported by Guo et al [[44], [45], [46]]. Vitamin C (VC) was adopted as the reference (positive) control. The experimental methods are detailed in Supplementary information S1.8–1.10.

### 2.11 Principal components analysis (PCA) of SDP

PCA was used to further analyze the multidimensional relationship between the yield, physicochemical properties, structural characteristics, and antioxidant activity of SDP. PCA reduced the dimensionality of high-dimensional data through orthogonal transformation and extracted principal components to characterize the data structure [47]. All indicators in the analysis were standardized.

### 2.12 Construction of a comprehensive scoring model for polysaccharides extraction techniques from S. Divaricata

The entropy weight method was applied to quantitatively determine the relative importance of different factors influencing the biological characteristics of SDP, thereby quantifying the indicators and determining the scoring criteria for each indicator, and the comprehensive evaluation index of polysaccharides extracted by each method was obtained. In order to solve the problem of insufficient subjective evaluation ability of the multi-index optimization method, information entropy was introduced in the optimization process to make the determination of weight coefficients more objective [48,49]. The central principle of the entropy-weight method is to assign objective weights in proportion to the degree of variability in the data [50]. Subsequently, technique for order preference by similarity to ideal solution (TOPSIS) was applied to compute each method’s relative closeness to the ideal solution, yielding an overall score and ranking. A Spearman correlation analysis was ultimately undertaken to evaluate correlations between the physicochemical measures and antioxidant activity, and all computations were completed using Pycharm 2025.1.1.1. Because the dimensions of each indicator differ, it is necessary to perform data standardization before further analysis. Common practice employs min–max normalization to constrain all measurements to the [0, 1] domain. Entropy values were calculated from the normalized data using Eqs. (2) and (3), and the proportion of each indicator among the samples was determined via Eqs. (4) and (5). Eq. (6) was used to calculate weights. Finally, the comprehensive score for each sample was computed in accordance with Eq. (7).

Positive indicators (the larger the better):

(2) $$\begin{array}{c} {x\prime }_{\mathrm{ij}}=\frac{x_{\mathrm{ij}}-min\left(x_{j}\right)}{max\left(x_{j}\right)-min\left(x_{j}\right)} \end{array}$$

Negative indicators (the smaller the better):

(3) $$\begin{array}{c} {x^{\prime }}_{\mathrm{ij}}=\frac{max\left(x_{j}\right)-x_{\mathrm{ij}}}{max\left(x_{j}\right)-min\left(x_{j}\right)} \end{array}$$

*x'_ij_* is the original value of the *i^th^* sample in the *j^th^* indicator.

(4) $$\begin{array}{c} P_{\mathrm{ij}}=\frac{{x^{\prime }}_{\mathrm{ij}}}{\sum_{i=1}^{n}{x^{\prime }}_{\mathrm{ij}}} \end{array}$$

*P_ij_* is the proportion of the *i^th^* sample under indicator j; n is the number of samples.

(5) $$\begin{array}{c} e_{j}=-k\sum_{i=1}^{n}p_{\mathrm{ij}}ln\left(p_{\mathrm{ij}}\right),k=\frac{1}{\ln\;n} \end{array}$$

*e_j_* ∈ [0,1] is the information entropy of indicator j; k is a constant whose value is to be determined.

(6) $$\begin{array}{c} \omega_{j}=\frac{1-e_{j}}{\sum_{j=1}^{m}1-e_{j}} \end{array}$$

*ω_j_* is the weight of indicator j.

(7) $$\begin{array}{c} S_{i}=\sum_{j=1}^{m}\omega_{j}{x\prime }_{\mathrm{ij}} \end{array}$$

*S_i_* is the comprehensive score of the *i^th^* sample.

A dual verification method combining algorithms and research results was used to determine positive and negative indicators, ensuring the objectivity and scientific nature of the indicator direction and avoiding subjective judgment bias. Based on the research of scholars [[51], [52], [53], [54], [55]], the positive indicators were set as polysaccharide yield, total sugar content, uronic acid content, DPPH, ABTS^+^, and ·OH, while the negative indicators were set as protein content and molecular weight. The direction of sulfate content, monosaccharide composition and triple helix structure was confirmed by calculating their correlation with the average antioxidant activity. The FTIR spectra of polysaccharides obtained through different techniques exhibited highly similar functional group patterns, leading to their exclusion from the evaluation criteria.

## 3 Results and discussion

### 3.1 Comparative analysis of extraction techniques for SDP

**Fig. 2:**
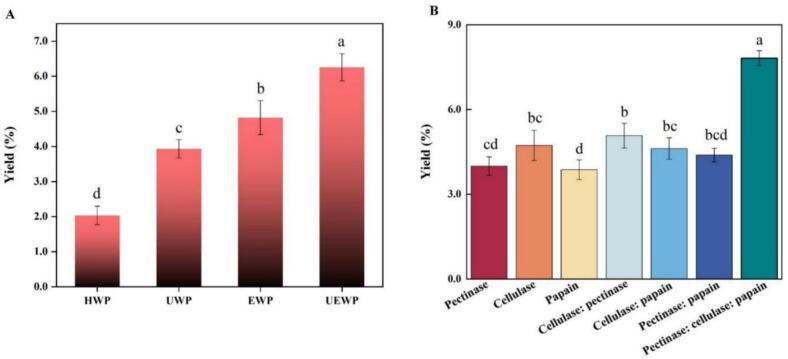
SDP yield (A) with different extraction methods (B) and different enzymes (wherein the ratio of composite enzymes is all 1:1or 1:1:1).

As illustrated in Fig. 2A, the yields of HWP, EWP, UWP, and UEWP were 2.03 ± 0.26 %, 3.93 ± 0.49 %, 4.82 ± 0.26 %, and 6.25 ± 0.39 %, respectively. The yield of UEWP was the highest among all samples, approximately twice that of HWP. The enzyme effectively breaks down cell walls and membranes at relatively low temperatures, aiding in the separation of the target polysaccharides and consequently increasing the yield. They disrupt cell walls and membranes, facilitating the release of polysaccharides and consequently enhancing the yield. Through cavitation formed in the solvent, ultrasonication facilitates the liberation, diffusion, and solubilization of polysaccharides [56]. Therefore, compared with HWP and EWP, the new auxiliary method is capable of greatly improving the yield of polysaccharides, and this finding aligns with results documented in numerous studies. Therefore, ultrasound-synergistic enzymatic extraction was chosen to refine the extraction procedure further.

### 3.2 Effect of different enzymes of SDP

As shown in Fig. 2B, the experiment assessed the influence of single enzymes and composite enzymes on the extraction efficiency of SDP. It was found that among the 7 different enzyme combinations, the mixture of cellulase: pectinase: papain (1:1:1) exhibited the highest polysaccharides yield. This may be attributed to the ability of cellulase and pectinase to degrade the cellulose framework and pectin matrix, thereby disrupting the cell wall structure and forming channels that facilitate polysaccharide release. Meanwhile, papain hydrolyzes the proteins in the glycoprotein complex, breaking the chemical bonds of polysaccharides and releasing both free and bound polysaccharides. The synergistic action of the three enzymes significantly outperforms the individual effect of a single enzyme or a binary combination [57].

### 3.3 Analysis of single-factor experiment results

**Fig. 3:**
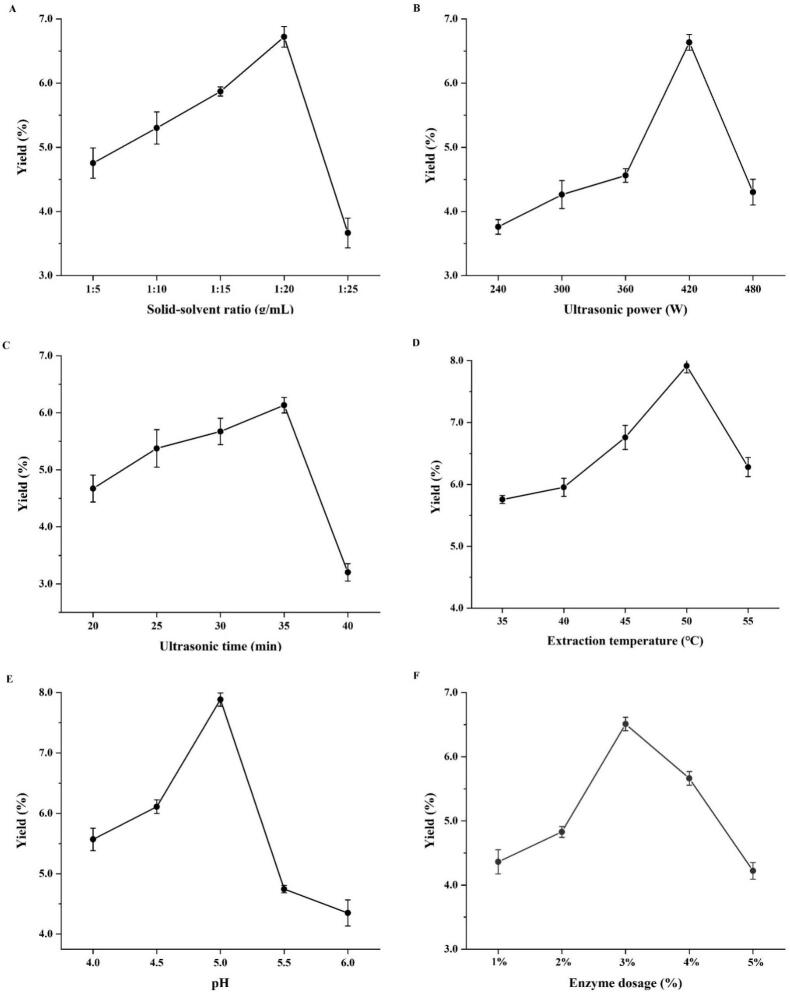
The impact of factors on the yield of SDP (A: solid-solvent ratio; B: ultrasonic power; C: ultrasonic time; D: extraction temperature; E: pH; F: enzyme dosage).

A series of single-factor experiments were performed to achieve optimal extraction efficiency. With the solid-solvent ratio continuously rising, the SDP yield initially increased and then declined (Fig. 3A). The highest extraction efficiency was achieved at a solid-solvent ratio of 1:20 g/mL, resulting in a yield of 6.72 ± 0.16 %. It is widely recognized that increasing the volume of extraction solvent allows for better contact with the sample, which in turn intensifies the concentration gradient between the intracellular and solution phases. This process increases the solubility of polysaccharides and facilitates their dissolution. In contrast, when the amount of solvent is relatively small, insufficient contact occurs, causing polysaccharides to fail to be fully extracted and thus resulting in a low yield. However, an excessive solvent volume leads to a reduction in substrate concentration, which diminishes the efficacy of the enzymatic process and interferes with the solubilization of polysaccharides [58,59].

A gradual increase in ultrasonic power from 240 W to 420 W was accompanied by a consistent rise in SDP yield (Fig. 3B). The yield reached its maximum of 6.64 ± 0.12 % at 420 W. When confined to a suitable range, elevating ultrasonic power intensifies cavitation, dismantles plant cell structures, and accelerates the solubilization of polysaccharides. Nevertheless, upon further increasing the power to 480 W, a decline in yield was observed. Excessive ultrasonic power induces intense cavitation, resulting in polysaccharide chain breakage and structural degradation, thereby decreasing the yield. Specifically, excessively high ultrasonic power may induce the cleavage or modification of polysaccharides molecules, leading to their structural disruption and thus a reduction in the polysaccharides yield. This result is corroborated by previous research reports [60].

The SDP yield significantly increased with the prolongation of ultrasonic time (Fig. 3C). The yield reached its maximum value of 6.13 ± 0.14 % when the time reached 35 min. This improvement may be attributed to the fact that an appropriately extended ultrasonic treatment time the solvent to penetrate more effectively into the raw material, thereby enhancing the dissolution and transfer of polysaccharides into the solvent, and ultimately enhancing the yield. However, when the time exceeded 40 min, a decrease in yield was observed. This may be attributed to the fact that excessively prolonged ultrasonic treatment induces degradation reactions in the solution, leading to depolymerization of polysaccharide chains and, consequently, a reduction in yield.

The SDP yield progressively increased as the temperature rises from 35 ℃ to 50 ℃, reaching its peak at 7.92 ± 0.11 % at 50 ℃ (Fig. 3D). After surpassing 50 ℃, incremental temperature increases were accompanied by a downward trend in yield. This may result from that elevated temperatures are capable of first inactivating the enzymes and even disrupting their spatial structure. Additionally, elevated temperatures may alter the structural integrity of polysaccharides and trigger partial degradation, thereby leading to a decline in yield.

The SDP yield increased with the increase in the pH value of the extraction solution (Fig. 3E). When the pH value of the extraction solution was 5.0, the yield reached the maximum of 7.88 ± 0.11 %, indicating that the composite enzymes exhibited the highest enzymatic activity and enzymatic hydrolysis efficiency under the weakly acidic condition of pH 5.0. As the pH of the solution increased, enzymatic activity diminished, leading to a reduction in yield. This may result from the fact that further elevation of pH can disrupt the spatial structure of enzymes (such as changes in hydrogen bonds and hydrophobic interactions), leading to abnormal enzyme conformation. Consequently, the ability of enzymes to catalyze the hydrolysis of cell walls is significantly impaired, and they cannot effectively disrupt the cell wall structure to release internal polysaccharides. Thus, a solution pH value of 5.0 was selected as the optimal pH for the reaction.

As depicted in Fig. 3F, the SDP yield rises initially with greater enzyme addition, but decreases thereafter. The highest yield of 6.51 ± 0.10 % is achieved when the enzyme addition is 3 %. This may be because, at low concentrations, enzymes bind with substrates to achieve a catalytic effect, and ultrasonic treatment further enhances the enzyme activity, thereby accelerating the breakdown of plant cell walls and the release of polysaccharides [61,62], thus improving the yield of SDP. However, as the enzyme concentration increases, the combination of enzymes and substrates gradually approaches saturation, resulting in no further increase in polysaccharides yield. In addition, excessively high enzyme concentration will cause enzymes to aggregate in the ultrasonic environment, reducing the extraction efficiency [63].

### 3.4 Screening of key factors by PB experiment

**Fig. 4:**
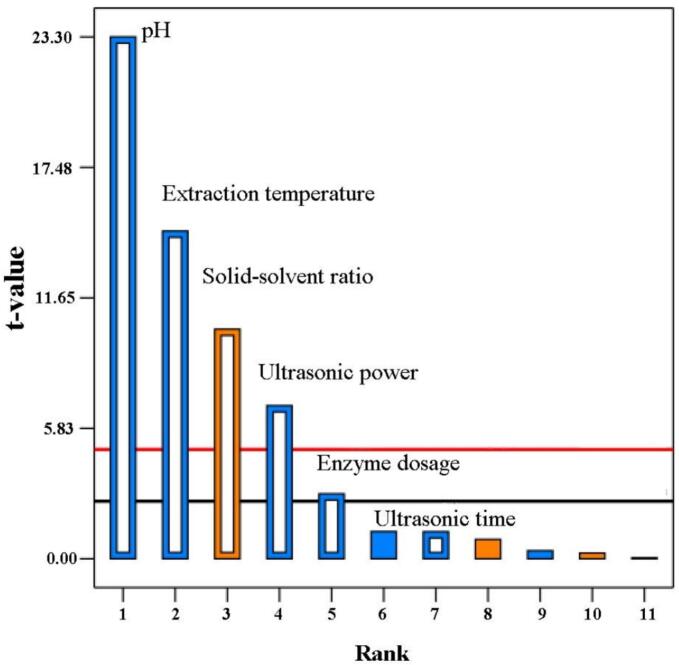
Pareto chart of single factor ranking (Note: Blue represents negative effects, yellow represents positive effects).

Following the single-factor experiment, the PB experiment was used to rank the six factors affecting the yield, including solid-solvent ratio, ultrasonic power, extraction temperature, ultrasonic time, pH, and enzyme dosage, with the aim of identifying the key factors and providing a basis for subsequent RSM optimization. As shown in Fig. 4, the factors that significantly affect the yield are pH, extraction temperature, solid-solvent ratio, ultrasonic power, and enzyme dosage. The results of the variance analysis for the PB model are provided in Supplementary information S2.1.

### 3.5 Analysis of RSM and SVR prediction model

#### 3.5.1 Analysis of RSM model

After identifying the solid-solvent ratio, extraction temperature, and pH as the key factors influencing the yield of UEWP in the PB experiment, the BBD was further applied to optimize the extraction procedure (Table 3).

**Table 3:** BBD for optimizing ultrasound-synergistic enzymatic extraction procedure of SDP.

**Run**	**A: Solid-solvent ratio (g/mL)**	**B:Extraction temperature (℃)**	**C:pH**	**Y: Yield (%)**
1	1:10	45	5.5	6.22 %
2	1:20	40	5	5.25 %
3	1:15	50	4.5	10.82 %
4	1:20	45	5.5	5.81 %
5	1:15	45	5	8.37 %
6	1:15	45	5	8.48 %
7	1:10	45	4.5	11.01 %
8	1:20	45	4.5	8.34 %
9	1:15	45	5	9.27 %
10	1:10	50	5	6.51 %
11	1:15	40	4.5	10.51 %
12	1:15	45	5	7.36 %
13	1:15	50	5.5	7.14 %
14	1:20	50	5	7.27 %
15	1:15	40	5.5	5.83 %
16	1:10	40	5	7.18 %
17	1:15	45	5	7.27 %

A regression analysis was performed on the obtained data, and the correlation equation between the response value and each variable value was calculated as follows:

Y (%) = 0.0815–0.0061*A + 0.0037*B − 0.0204*C + 0.0067*AB + 0.0072*AC + 0.0025*BC − 0.0109*A^2^- 0.0051*B^2^ + 0.0093*C^2^.

In the formula, Y represents the response value (predicted yield of SDP), A denotes the solid-solvent ratio, B stands for extraction temperature, and C indicates pH value. The experimental data were analyzed by variance analysis, and the results are shown in Table 4.

**Table 4:** RSM model ANOVA analysis.

**Source**	**Sum of squares**	**df**	**Mean square**	**F-value**	***p*-value**	
Model	0.0051	9	0.0006	10.85	0.0024	*
A-Solid-solvent ratio	0.0003	1	0.0003	5.67	0.0488	*
B-Extraction temperature	0.0001	1	0.0001	2.13	0.1882	–
C-pH	0.0033	1	0.0033	63.87	< 0.0001	**
AB	0.0002	1	0.0002	3.49	0.1041	–
AC	0.0002	1	0.0002	3.94	0.0875	–
BC	0	1	0	0.482	0.5099	–
A^2^	0.0005	1	0.0005	9.62	0.0173	*
B^2^	0.0001	1	0.0001	2.1	0.1905	–
C^2^	0.0004	1	0.0004	7.08	0.0325	*
Residual	0.0004	7	0.0001			
Lack of Fit	0.0001	3	0	0.3895	0.7677	–
Pure Error	0.0003	4	0.0001			
Cor Total	0.0054	16				

** Indicates highly significant (*p* < 0.001); * indicates significant (*p* < 0.05); – indicates not significant.

**Fig. 5:**
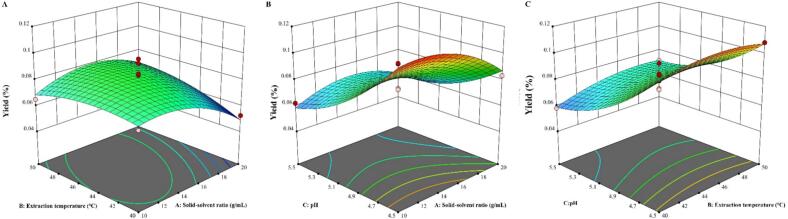
3D response surface plot of pairwise interactions between factors (A: AB interaction; B:AC interaction; C: BC interaction).

The results of variance analysis for the RSM experiment are presented in Table 4. The RSM is significant (*p* < 0.05), and the lack-of-fit term is not significant (*p* > 0.05). From the analysis results, in the quadratic terms, both the A and C have significant effects on Y, while all cross terms have no significant effect on Y. According to the F-values, the relative influence of the variables on the response (Y) followed the order: C > A > B. Fig. 5 displays distinct geometric patterns, reflecting varying degrees of interaction among the extraction variables. Among them, the surfaces in Fig. 5A and Fig. 5B are more pronounced, whereas Fig. 5C appears relatively flat, indicating that the interactions between AB and AC are stronger than that between BC. These results are all able to represent the relationship between each response and the independent variables, indicating that the model is able to well fit the experimental prediction results. In addition, the coefficient of determination R^2^ = 0.9331, the adjusted R^2^ = 0.8471, and the coefficient of variation (C.V.) = 9.19 %. All R^2^ values were within the acceptable range, and the coefficient of variation is low, suggesting that the model can be used to predict the yield of SDP.

#### 3.5.2 Analysis of SVR model

**Fig. 6:**
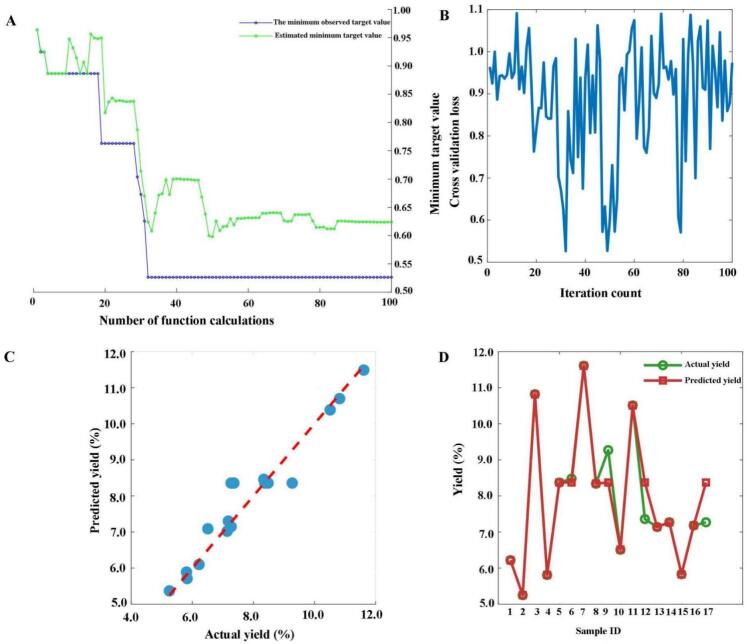
Prediction results of the SVR model (A: Minimum target value and number of function evaluations; B: Bayesian optimization process; C: Linear regression; A: Actual value vs. Predicted value).

ML has become a robust method for modeling and optimizing extraction procedure, as it can create models and evaluate multiple responses simultaneously. SVR, a regression algorithm developed based on the idea of Support Vector Machine, is widely used in dealing with prediction problems involving nonlinear, small-sample, and high-dimensional data. Compared to the RSM model, the SVR model exhibits higher accuracy in fitting experimental responses, predicting biochemical processes, and modeling, making it an alternative to complex nonlinear multivariate models. The network was trained using Bayesian algorithms (Fig. 6), resulting in an R^2^ = 0.9353, RMSE = 0.0043, MSE = 0.000018, and MAE = 0.0020 for the model, indicating its excellent predictive and optimization capabilities. The model predicts the optimal extraction parameters as a solid-solvent ratio of 1:12.51 g/mL, extraction temperature of 44.74 °C, pH = 4.5, and a predicted yield of  12.03%.

#### 3.5.3 Validation of RSM and SVR models

The SVR model achieved a higher R^2^ than the RSM model, reflecting its enhanced predictive performance. For the controllability of experimental operations, the predicted optimal conditions were slightly modified. The RSM model predicted a solid-solvent ratio of 1:11 g/mL, extraction temperature of 43 ℃, and pH = 4.5; the SVR prediction model predicted a solid-solvent ratio of 1:13 g/mL, extraction temperature of 45 ℃, and pH = 4.5. Through comparison with the RSM and SVR models, the optimization performance and verification results are shown in Table 5. Although the optimal ranges predicted by RSM and SVR are comparable, SVR achieves higher predictive accuracy with lower errors when fitted to the experimental data. The final measured yield (11.82 %) closely matches the SVR prediction, indicating greater reliability within the experimental domain. Model optimization comparison results are shown in the supplementary information S2.2.

**Table 5:** Validation results of RSM and SVR models.

**Model**	**Solid-solvent ratio** **(g/mL)**	**Extraction temperature (℃)**	**pH**	**Prediction yield**	**Actual yield**
RSM	1:11.42	43.23	4.5	11.70 %	11.15 %
SVR	1:12.51	44.74	4.5	12.03 %	11.82 %

### 3.6 Physicochemical properties of SDP

Table 6 displays the physicochemical properties of SDP obtained through various extraction techniques. The findings revealed a progressive increase in total sugar content with the refinement of extraction techniques. Notably, the total sugar content of UEWP peaked at 74.03 ± 0.28 %, indicating the superior efficacy of ultrasound-synergistic enzyme extraction in enhancing polysaccharides concentration. Conversely, UEWP exhibited a low protein content of 8.93 ± 0.04 %, attributed to enzymatic degradation for protein removal. The application of ultrasound further augmented enzyme efficiency through physical mechanisms, underscoring its potential for minimizing protein residues. The uronic acid content of UEWP (14.15 ± 0.67 %) surpassed that of other methods, owing to the gentle enzymatic conditions and ultrasonic disruption promoting uronic acid enrichment. Moreover, sulfate content decreased progressively with technological advancements in extraction (from HWP to UEWP), with HWP at 26.05 ± 0.55 % and UEWP at 14.43 ± 0.24 %. This is because the synergistic effect of ultrasound cavitation and enzymatic cleavage enhances the disruption of the cell wall/middle lamina and mass transfer processes, promoting the release of acidic pectin-like domains, while also reducing the co-precipitation of polysaccharide-protein complexes. This trend is consistent with previous observations of ultrasound-extracted polysaccharides, which often involve increased yield accompanied by a decrease in apparent molecular weight, enrichment of acidic domains, and removal of proteins [[64], [65], [66]]. These changes are typically associated with stronger free radical scavenging and reducing capabilities.

**Table 6:** Contents related to physicochemical properties of SDP.

**Source**	**Total sugar (%)**	**Protein (%)**	**Uronic acid(%)**	**Sulfate (%)**
HWP	67.55 ± 2.88	17.65 ± 1.34	5.38 ± 0.15	26.05 ± 0.55
EWP	70.05 ± 0.38	15.03 ± 0.05	8.83 ± 0.67	19.44 ± 0.12
UWP	70.87 ± 0.12	12.38 ± 0.13	7.06 ± 0.55	16.06 ± 0.25
UEWP	74.03 ± 0.28	8.93 ± 0.04	14.15 ± 0.67	14.43 ± 0.24

In summary, different extraction techniques markedly affected the physicochemical profiles of SDP. The ultrasound-synergistic enzymatic extraction exhibited superior performance in enhancing total sugar content, reducing protein and sulfate content, and enriching uronic acid levels. These results offer crucial data support for investigating structure–activity relationships and identifying optimal extraction techniques for SDP.

### 3.7 Analysis of SDP structural characteristics

#### 3.7.1 Analysis of FTIR spectroscopy results

**Fig. 7:**
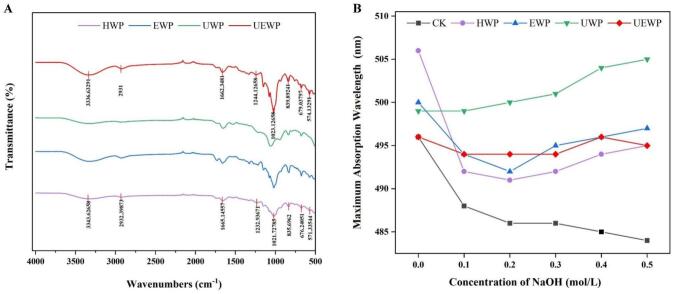
FTIR spectra(A) and triple helix structure(B) of SDP.

FTIR, an effective technique for probing the functional groups of polysaccharides, has seen broad application. As shown in Fig. 7A, the infrared spectra obtained from the four extraction methods in the 4000–500 cm^−1^ region exhibit high similarity, indicating similar functional group structures. Specifically, the abundant hydroxyl groups in polysaccharides molecules produce a strong and broad absorption peak in the 3200–3600 cm^−1^ region, which broadens due to hydrogen bonding interactions between hydroxyl groups. The 2800–3000 cm^−1^ region reflects C–H vibrations of the polysaccharide ring skeleton, a signature of the carbon–hydrogen framework. C=O stretching in carboxyl groups (–COOH) at 1600–1700 cm^−1^ confirms that all four polysaccharides contain uronic acid, in agreement with earlier reports. The sulfate ester group (–O–SO_3_^−^) in sulfated polysaccharides exhibits characteristic absorption at 1200–1260 cm^−1^, corresponding to the asymmetric stretching vibration of S=O, which confirms the presence of sulfate groups. The absorption observed at 1244 cm^−1^ suggests that the polysaccharides are sulfated, with sulfate groups linked to the hydroxyl groups of sugar rings through ester bonds. The range of 1000–1100 cm^−1^ serves as the characteristic fingerprint for polysaccharides. Signals centered at 1020–1050 cm^−1^ primarily reflect C–O–C glycosidic stretching together with C–O vibrations in pyranose rings, consistent with pyranose motifs. Most natural polysaccharides exhibit a pyranose configuration. The absorption near 840 cm^−1^, assigned to S–O–C stretching, along with the S=O band at 1244 cm^−1^, verifies sulfate substitution in the polysaccharides. Absorption in the 600–800 cm^−1^ region is usually related to the backbone vibration of polysaccharides (such as bending of pyranose rings). Infrared spectroscopy indicates that all four polysaccharides contain pyranose rings, sulfate groups, and uronic acid, and may be acidic polysaccharides.

#### 3.7.2 Analysis of Congo red

The conformational features of polysaccharide chains in aqueous solution are frequently examined using the Congo red assay. Based on different concentrations of NaOH solution system, the red shift of the maximum absorption wavelength (λmax) after polysaccharide combined with Congo red was observed to infer the structural characteristics of the polysaccharide sample [67]. As shown in Fig. 7B, the red shift of HWP is the most obvious, followed by UWP, while the absorption wavelengths of EWP and UEWP do not show a significant downward trend, thus it can be judged that EWP and UEWP do not have a triple helix structure. The addition of mixed enzymes likely modifies polysaccharide molecular structures, a finding consistent with the results reported by Chen et al. [68]. Huang et al. [69] believe that polysaccharides without a triple helix structure are able to exhibit higher biological activity. This may be because the destruction of the triple helix structure by ultrasound is conducive to the exposure of active sites and the improvement of biological activity, which can also prove that UEWP has the highest antioxidant activity.

#### 3.7.3 Analysis of molecular weight

As a determinant of numerous properties, polysaccharide molecular weight plays a pivotal role in structural evaluation. As presented in Table 7, polysaccharides extracted using hot water have the highest molecular weight, whereas those obtained through ultrasonic-assisted enzymatic extraction possess the lowest. The phenomenon can be ascribed to cavitation induced by ultrasound that compromises the integrity of polysaccharides, splitting larger chains and producing smaller molecular-weight components. Additionally, the synergistic effect of compound enzymes can further disrupt the polysaccharides structure more extensively. This finding is broadly in agreement with the conclusions of Cheung and Colussi [70,71], further confirming that polysaccharide molecular weight varies with the extraction conditions. Previous investigations [72,73] have indicated that reduced molecular weight correlates with enhanced bioactivity in polysaccharides, consistent with our antioxidant results: UEWP possesses the lowest molecular mass and the strongest antioxidant activity. No significant differences in polydispersity coefficients were observed among polysaccharides obtained by different extraction methods in this study, indicating similar aggregation levels.

**Table 7:** Molecular weights of SDP.

**Source**	**Mn (×10^4^ Da)**	**Mw (×10^4^ Da)**	**Polydispersity (Mw/Mn)**
HWP	108.3166	512.8860	4.735
EWP	96.7057	429.0557	4.437
UWP	69.7627	319.6996	4.583
UEWP	17.1290	86.5000	5.050

#### 3.7.4 Analysis of monosaccharide composition

Analyzing the monosaccharide composition of polysaccharides is foundational for elucidating their structural features and physicochemical properties. The monosaccharide compositions of the polysaccharides extracted by different treatment are the same, all including arabinose (Ara), rhamnose (Rha), galactose (Gal), glucose (Glc), xylose (Xyl), mannose (Man), galacturonic acid (Gal-UA), and glucuronic acid (Glc-UA), but their compositional proportions differ **(**Table 8**)**. The monosaccharide composition of HWP is Ara (10.30 %): Rha (1.29 %): Gal (10.74 %): Glc (68.66 %): Xyl (0.61 %): Man (0.51 %): Gal-UA (6.45 %): Glc-UA (1.44 %). The monosaccharide composition of EWP is Ara (9.07 %): Rha (0.84 %): Gal (11.46 %): Glc (71.33 %): Xyl (0.45 %): Man (0.54 %): Gal-UA (4.87 %): Glc-UA (1.44 %). The monosaccharide composition of UWP is Ara (35.40 %): Rha (1.73 %): Gal (54.40 %): Glc (3.12 %): Xyl (0.82 %): Man (1.09 %): Gal-UA (1.45 %): Glc-UA (1.99 %). The monosaccharide composition of UEWP is Ara (8.82 %): Rha (0.64 %): Gal (12.03 %): Glc (75.35 %): Xyl (0.27 %): Man (0.84 %): Gal-UA (0.51 %): Glc-UA (1.31 %). Ara, Gal, and Glu are identified as the main monosaccharide components of SDP accounting for more than 85 % in total. The variation in monosaccharide content may result from ultrasound, which, through cavitation and shear forces, breaks down the extended chains of polysaccharides and facilitates the cleavage of glycosidic bonds. Simultaneously, enzymes alter the molecular structure of polysaccharides, leading to further effects.

**Table 8:** Monosaccharide compositions of SDP.

**Source**	**Ara**	**Rha**	**Gal**	**Glc**	**Xyl**	**Man**	**Gal-UA**	**Glc-UA**
HWP	10.30 %	1.29 %	10.74 %	68.66 %	0.61 %	0.51 %	6.45 %	1.44 %
EWP	9.07 %	0.84 %	11.46 %	71.33 %	0.45 %	0.54 %	4.87 %	1.44 %
UWP	35.40 %	1.73 %	54.40 %	3.12 %	0.82 %	1.09 %	1.45 %	1.99 %
UEWP	8.82 %	0.64 %	12.03 %	75.35 %	0.27 %	0.84 %	0.51 %	1.31 %

#### 3.7.5 Morphological feature analysis by SEM

**Fig. 8:**
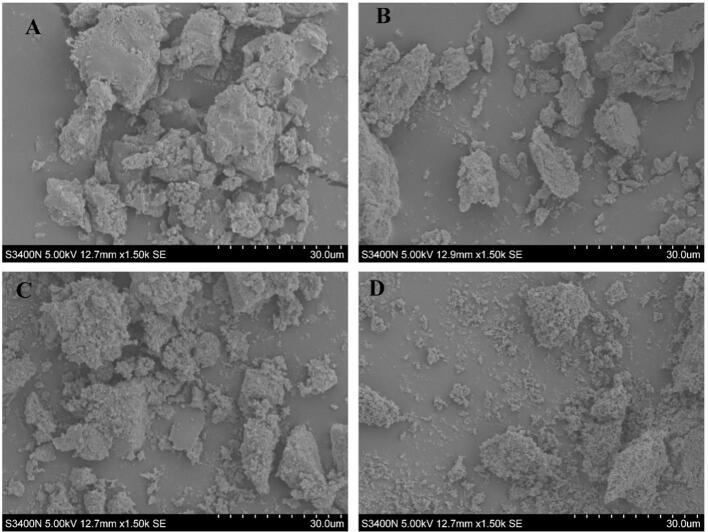
SEM images of different extraction methods of SDP (A: HWP; B: EWP; C: UWP; D: UEWP).

As shown in Fig. 8, the polysaccharides obtained by HWP appear as dense blocks with relatively smooth surfaces. This may be because the prolonged action of high-temperature water has the ability to dissolve the polysaccharides, but at the same time, it can cause thermal denaturation, aggregation, and dehydration of some polysaccharides chains. During the drying process, this aggregation tendency is exacerbated, forming larger, tightly packed block structures. Compared with HWP, EWP exhibits a looser structure with a higher degree of fragmentation, presenting smaller flakes, particles, or irregular fragments, with a rougher surface and more pores. The addition of enzymes is able to specifically hydrolyze non-polysaccharides components in plant cell walls and/or some glycosidic bonds. This disrupts the rigid configuration of the cell wall and the bonding between polysaccharide chains, making the polysaccharides encapsulated within more easily released. During the drying process, these smaller fragments and the gaps generated by enzymatic hydrolysis appear as a looser, fragmented, and porous structure. UWP shows a honeycomb or sponge-like structure. Fibrous networks or loose agglomerates formed by the aggregation of finer particles may also be observed. Its structure is looser than that of HWP. The surface of UEWP is loose, fragmented, porous, even flocculent, and irregularly distributed. The cavitation generated by ultrasound markedly strengthens contact between enzymes and their substrates, thereby expediting enzymatic decomposition. Meanwhile, the mechanical force of ultrasound itself breaks down cells and molecules, working in synergy with the chemical cleavage effect of enzymes. Under this dual action, the cell walls and the structure of the original polysaccharides are broken down most thoroughly. The released polysaccharides may have a lower molecular weight and weaker aggregation, and after drying, they exhibit the loosest structure with the highest degree of fragmentation and most well-developed pores.

### 3.8 Analysis of in vitro antioxidant activity of SDP

**Fig. 9:**
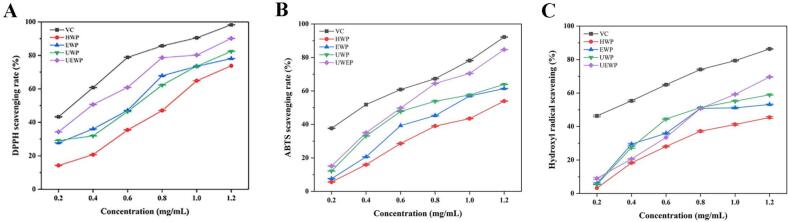
Antioxidant activity results of SDP (A: DPPH; B: ABTS^+^; C: Hydroxyl radical).

The DPPH radical scavenging activity of SDP extracts obtained through various methods is presented in Fig. 9A. All polysaccharide samples demonstrated potent antioxidant properties, achieving scavenging rates exceeding 80 %, while clearly displaying a concentration-dependent response. Fig. 9B illustrates the ABTS^+^ radical scavenging performance of the SDP samples. As the concentration of polysaccharides increased from 0.2 mg/mL to 0.8 mg/mL, a marked enhancement in scavenging efficiency was observed. Notably, the UEWP extraction method yielded samples with superior ABTS^+^ radical neutralizing capability compared to other techniques. The hydroxyl radical, a highly reactive oxygen species generated within biological systems, readily interacts with cellular biomolecules, potentially triggering apoptosis or tissue damage. Mirroring the trends observed in DPPH and ABTS^+^ assays, the radical scavenging efficiency shows a dose-dependent response, with higher sample concentrations yielding progressively greater neutralization rates (Fig. 9C). In summary, compared to the HWP, UWP, and EWP, UEWP demonstrates enhanced antioxidant activity, possibly due to the combined influence of enzymes and ultrasound, leading to structural modifications in the polysaccharides that produce a beneficial effect.

### 3.9 Analysis of PCA results

**Fig. 10:**
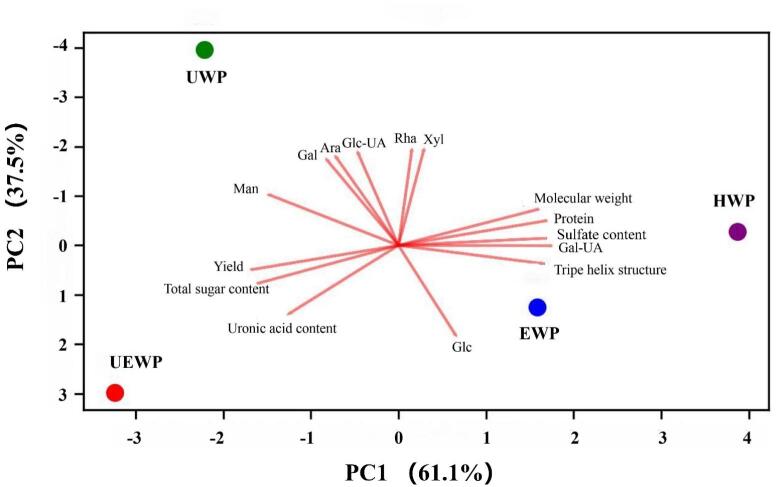
PCA score plot of SDP obtained by different extraction methods.

PCA was employed to gain a comprehensive overview of the relationships and variations among the four polysaccharide extracts. In aggregate, the first two PCs described 98.6 % of the variance present in the dataset, validating the applicability of the PCA model for characterizing differences between samples. The score plot (Fig. 10) showed that samples obtained via traditional methods (HWP, EWP) and modern extraction methods (UWP, UEWP) were clearly separated along PC1. UWP exhibited distinct separation along PC2, indicating that it has unique characteristics compared with other extraction methods. In contrast, UWP and UEWP were not only clearly separated from the traditional extraction methods but also distinguishable from each other. This separation suggests that ultrasonic treatment and ultrasound-synergistic enzymatic treatment significantly alter the properties of the extracted polysaccharides. In the plot, the vector length of variables represents their contribution to the principal components, with a longer vector indicating a greater contribution. Molecular weight, triple helix structure, Man, Glc, yield, total sugar content, and uronic acid content exerted a more significant influence on the PCs.

### 3.10 Establishment of comprehensive evaluation model for SDP extraction methods

**Fig. 11:**
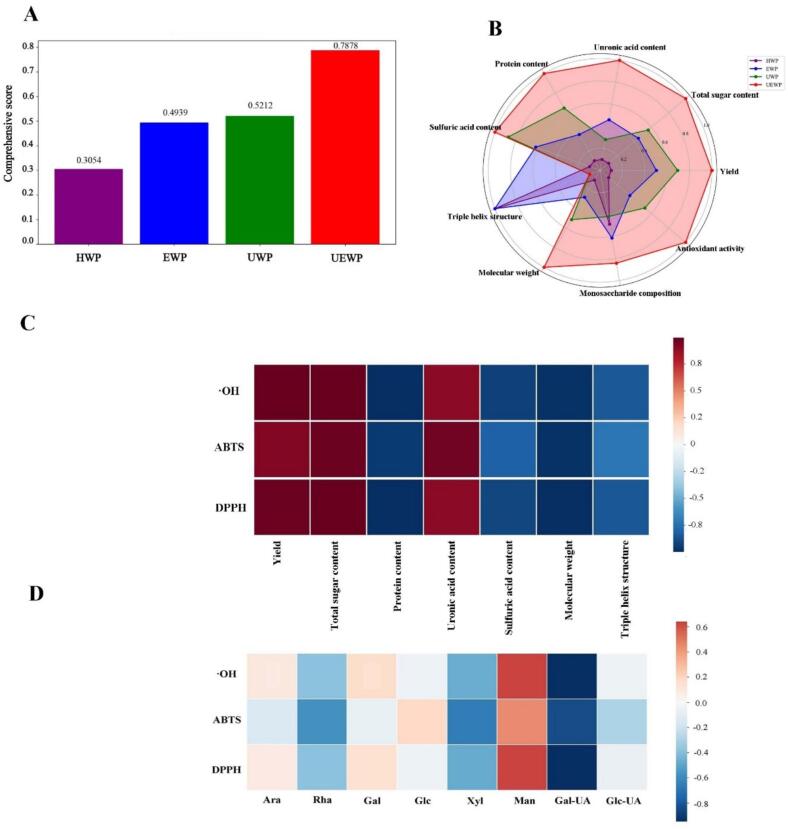
Comprehensive evaluation indexes of SDP by different extraction methods (A: Comprehensive scores; B: Indicator performance radar chart; C: Spearman correlation heatmap; D: Heat map of monosaccharide composition-antioxidant activity correlation).

The entropy weight method is an objective evaluation technique that quantifies the effective information in known data and assigns index weights through the calculation of the information entropy of indicators, thus reducing biases caused by human factors. The ranking of comprehensive scores (Fig. 11A) was: UEWP (0.7878) > UWP (0.5212) > EWP (0.4939) > HWP (0.3054), indicating that ultrasonic-assisted enzymatic extraction represents the optimal method. The visualization in Fig. 11B provides a multi-dimensional feature analysis, clearly demonstrating the balanced superiority of UEWP in all key indicators, while the feature profiles of other methods are relatively limited. Fig. 11C further illustrates that the yield, polysaccharide content, and uronic acid content were positively correlated with antioxidant activity, while the protein content, sulfate content, molecular weight, and triple helix structure were negatively correlated with antioxidant activity. These relationships suggest that smaller molecular size and greater purity enhance antioxidant capacity. Studies have also confirmed that the small molecular weight and elevated uronic acid content of polysaccharides are generally associated with stronger antioxidant activity [74,75]. Man was strongly positively correlated with antioxidant activity, while Gal-UA was negatively correlated (Fig. 11D). Integrating the entropy-weight analysis (Supporting information S2.4) shows that the informational contribution of physicochemical and structural indices differs across extraction methods. Notably, Man (0.074), uronic acid content (0.059), and molecular weight (0.058) carry the highest weights, indicating strong discriminative power in cross-method comparisons. Consistent with the Pearson correlation results, these three indices exhibit significant positive or negative associations with antioxidant capacity, suggesting that they are not only information-rich but also key determinants of antioxidant activity.

Our findings support the conclusions of earlier studies that highlight ultrasound-synergistic enzymatic extraction as an effective method for isolating polysaccharides from various plant sources, further endorsing its potential for broader use in polysaccharide research [[76], [77], [78]]. Ultrasonic cavitation aids in breaking down cell walls and facilitating mass transfer, while enzymatic hydrolysis targets the degradation of structural components, which leads to enhanced extraction efficiency and improved retention of bioactive compounds. On the one hand, the lower molecular weight of UEWP may facilitate better absorption and interaction with free radicals, thereby enhancing antioxidant activity. On the other hand, although ultrasound may have disrupted the helical structure of polysaccharides, it exposes more active sites, which in turn increases the uronic acid content. This is precisely the value of process screening. This method overcomes subjective weighting bias, highlights key indicators with high variability, and enables objective ranking of the four extraction processes.

## 4 Conclusion

Compared with traditional process optimization methods that focus solely on the content of a single active component, multi-index comprehensive evaluation has emerged as a key trend in the quality assessment of TCMs. SDP were extracted in this work through ultrasound-synergistic enzymatic. The extraction process was optimized using RSM and the ML method, based on the results of the single-factor experiment. Results revealed that ML algorithms exhibit higher accuracy in handling complex nonlinear relationships and enable more precise prediction of polysaccharides yield. A novel strategy for the intelligent optimization of extraction processes is provided by this approach. Furthermore, a comparative evaluation assessment of physicochemical properties, structural characteristics, and antioxidant activities of SDP extracted using different techniques revealed that UEWP has significant advantages in many aspects. This study not only shows that ultrasound-synergistic enzymatic extraction is an efficient method for extracting SDP, which can improve the polysaccharide extraction efficiency and antioxidant activity, but more importantly, it reveals that uronic acid content, molecular weight and Man are key indicators affecting the antioxidant activity of SDP, providing a theoretical basis and practical guidance for the directional preparation of functional polysaccharides.

## CRediT authorship contribution statement

**Zeyu Li:** Writing – original draft, Validation, Software, Methodology. **Qian Li:** Writing – review & editing, Supervision. **Chaogui Hu:** Validation, Methodology. **Feng Yang:** Validation, Formal analysis. **Fengxia Guo:** Supervision, Formal analysis.

## Declaration of competing interest

The authors declare that they have no known competing financial interests or personal relationships that could have appeared to influence the work reported in this paper.
